# Developmental Trajectories of Body Mass Index Among Japanese Children and Impact of Maternal Factors during Pregnancy

**DOI:** 10.1371/journal.pone.0051896

**Published:** 2012-12-13

**Authors:** Chiyori Haga, Naoki Kondo, Kohta Suzuki, Miri Sato, Daisuke Ando, Hiroshi Yokomichi, Taichiro Tanaka, Zentaro Yamagata

**Affiliations:** 1 Department of Health Sciences, Interdisciplinary Graduate School of Medicine and Engineering, University of Yamanashi, Yamanashi, Japan; 2 Department of Health Economics and Epidemiology Research, University of Tokyo School of Public Health, Tokyo, Japan; 3 Department of Physical Education, National Defense Academy, Kanagawa, Japan; 4 Department of Environmental and Occupational Health, Faculty of Medicine, Toho University, Tokyo, Japan; **No. of Participants**; **Group 1 Stable Thin**; **Group 2 & 3 Stable Average**; **Group 4 Progressive Overweight**; **Group 5 Progressive Obesity**; **No. of Participants**; **Group 1 Stable Thin**; **Group 2 & 4 Stable Average**; **Group 3 Progressive Average**; **Group 5 Progressive Overweight**; **Group 6 Progressive Obesity**; Pennington Biomedical Research Center/LSU, United States of America

## Abstract

**Background:**

The aims of this study were to 1) determine the distinct patterns of body mass index (BMI) trajectories in Japanese children, and 2) elucidate the maternal factors during pregnancy, which contribute to the determination of those patterns.

**Methodology/Principal Findings:**

All of the children (1,644 individuals) born in Koshu City, Japan, between 1991 and 1998 were followed in a longitudinal study exploring the subjects’ BMI. The BMI was calculated 11 times for each child between birth and 12 years of age. Exploratory latent class growth analyses were conducted to identify trajectory patterns of the BMI z-scores. The distribution of BMI trajectories were best characterized by a five-group model for boys and a six-group model for girls. The groups were named “stable thin,” “stable average,” “stable high average,” “progressive overweight,” and “progressive obesity” in both sexes; girls were allocated to an additional group called “progressive average.” Multinomial logistic regression found that maternal weight, smoking, and skipping breakfast during pregnancy were associated with children included in the progressive obesity pattern rather than the stable average pattern. These associations were stronger for boys than for girls.

**Conclusions/Significance:**

Multiple developmental patterns in Japanese boys and girls were identified, some of which have not been identified in Western countries. Maternal BMI and some unfavorable behaviors during early pregnancy may impact a child’s pattern of body mass development. Further studies to explain the gender and regional differences that were identified are warranted, as these may be important for early life prevention of weight-associated health problems.

## Introduction

Childhood obesity is associated with cardiovascular [1], [2], endocrine [3], [4], and respiratory diseases [5] in childhood, and these risks are likely to track into adulthood [6]. These associations suggest that physical development in early childhood can strongly determine health risks during adulthood. To date, most epidemiologic studies examining obesity have focused on physical attributes at a single time point [7], [8], and such studies often provide misleading data because they do not take into account physical attributes that vary over time during the natural development of children. Recent developments in statistical techniques that allow the analysis of longitudinal data generated from repeated measurements have enabled researchers to identify distinctive developmental “patterns” in an exploratory manner. Hoekstra et al. applied a novel latent-class growth-modeling approach [9] to longitudinal data in Holland (n = 336), and identified 3 distinct trajectories of body mass index (BMI) in individuals between the ages of 13 and 42 years, namely, the “normative,” “progressively overweight,” and “progressively overweight but stabilizing” trajectories. These risks were linked to differential cardiovascular risks in adulthood [10].

There have also been a few studies that have explored BMI trajectories in early childhood. A study in the United States monitored children aged 9–16 years and found 4 developmental patterns: “constant obesity,” “gradual obesity,” “obesity followed by recovery of normal weight,” and “never obese.” Another study in the United States identified 3 patterns among children up to 12 years old [11], [12], and a Canadian study tracked children aged 2–8 years and detected 3 growth patterns in boys and 4 in girls [13]. However, all of these studies were based on observations made exclusively in Western countries, making the results of less relevance to Asian populations. The results are most pertinent to Western populations since body mass and growth patterns can vary greatly depending on race/ethnicity [14]. For example, BMI in Asians is more likely to be lower than that of individuals from the West [15]. These regional differences may be attributable to variations in diets (i.e., higher calories and more fat in Western diets) [16], [17].

Although the determinants of these differential growth patterns are largely unknown, environmental exposures *in utero* [18], [19] and after birth, including maternal health and health behaviors during pregnancy and the child’s socio-economic status and lifestyle (e.g., diet, physical exercise) have been suggested as possible determinants of differential developmental patterns [11]–[13]. Therefore, the aims of this study were to 1) determine the distinct patterns of BMI trajectories in Japanese children from birth through 12 years of age with an exploratory approach, and 2) elucidate maternal factors, during pregnancy, which may contribute to the determination of those patterns. We hypothesized that there may be more variations among the low BMI patterns in the Japanese data, in addition to the normal and obese patterns that were previously identified by studies carried out in Western regions [11]–[13]. This is the first study identifying the long-term BMI trajectory patterns of children in an Asian country.

## Results

### BMI Trajectories

Maternal ages ranged from 16 to 42 years (mean, 28.9 years) for boys and from 18 to 44 years (mean, 28.9) for girls; paternal ages ranged from 17 to 48 years (mean, 32.0) for boys and from 18 to 56 years (mean, 31.9) for girls (Table 1).

**Table 1:** Characteristics of children by body mass index trajectories, Koshu City, Japan, 1991–1998.

	Boys										Girls											
	No. of Participants		Goup 1 Stable Thin		Group 2 & 3 Stable Average		Group 4 Progressive Overweight		Group 5 Progressive Obesity		No. of Participants		Group 1 Stable Thin		Group 2 & 4 Stable Average		Group 3 Progressive Average		Group 5 Progressive Overweight		Group 6 Progressive Obesity	
Variables	n	(%)	n	(%)	n	(%)	n	(%)	n	(%)	n	(%)	n	(%)	n	(%)	n	(%)	n	(%)	n	(%)
Year of birth																						
1991	107	(100%)	14	(13.1%)	80	(74.8%)	10	(9.3%)	3	(2.8%)	111	(100%)	19	(17.1%)	65	(58.6%)	6	(5.4%)	19	(17.1%)	2	(1.8%)
1992	111	(100%)	10	(9.0%)	87	(78.4%)	11	(9.9%)	3	(2.7%)	105	(100%)	8	(7.6%)	69	(65.7%)	16	(15.2%)	9	(8.6%)	3	(2.9%)
1993	107	(100%)	5	(4.7%)	89	(83.2%)	6	(5.6%)	7	(6.5%)	93	(100%)	17	(18.3%)	52	(55.9%)	11	(11.8%)	8	(8.6%)	5	(5.4%)
1994	95	(100%)	10	(10.5%)	77	(81.1%)	6	(6.3%)	2	(2.1%)	136	(100%)	17	(12.5%)	84	(61.8%)	15	(11.0%)	14	(10.3%)	6	(4.4%)
1995	110	(100%)	11	(10.0%)	81	(73.6%)	13	(11.8%)	5	(4.5%)	112	(100%)	16	(14.3%)	70	(62.5%)	9	(8.0%)	12	(10.7%)	5	(4.5%)
1996	90	(100%)	11	(12.2%)	66	(73.3%)	10	(11.1%)	3	(3.3%)	99	(100%)	11	(11.1%)	66	(66.7%)	9	(9.1%)	10	(10.1%)	3	(3.0%)
1997	113	(100%)	19	(16.8%)	74	(65.5%)	14	(12.4%)	6	(5.3%)	91	(100%)	12	(13.2%)	58	(63.7%)	5	(5.5%)	15	(16.5%)	1	(1.1%)
1998	92	(100%)	14	(15.2%)	70	(76.1%)	6	(6.5%)	2	(2.2%)	72	(100%)	13	(18.1%)	46	(63.9%)	7	(9.7%)	5	(6.9%)	1	(1.4%)
Total	825	(100%)	94	(11.4%)	624	(75.6%)	76	(9.2%)	31	(3.8%)	819	(100%)	113	(13.8%)	510	(62.3%)	78	(9.5%)	92	(11.2%)	26	(3.2%)
Maternal age (years): means (SD)	824		28.5	(3.84)	28.7	(4.29)	29.5	(4.13)	28.5	(4.20)	810		29.2	(4.58)	28.6	(4.28)	29.5	(4.42)	30.0	(4.18)	30.3	(5.24)
Maternal Body Mass Index (kg/m^2^): means (SD)	720		19.9	(2.60)	20.7	(2.61)	22.2	(3.47)	22.7	(3.30)	694		19.5	(2.38)	20.6	(2.62)	21.3	(3.21)	21.5	(2.87)	23.7	(4.55)
Maternal educational attainment (more than high school)	297	(52.1%)	36	(56.3%)	228	(52.9%)	23	(43.4%)	10	(45.5%)	301	(52.3%)	38	(46.3%)	196	(54.6%)	29	(55.8%)	30	(47.6%)	8	(40.0%)
Maternal parity (first birth)	322	(39.1%)	38	(40.4%)	243	(39.0%)	25	(34.2%)	16	(48.5%)	357	(43.6%)	47	(41.2%)	234	(46.3%)	35	(42.7%)	32	(35.2%)	9	(34.6%)
Child’s Body Mass Index (kg/m^2^) at birth; means (SD)	812		12.6	(1.15)	12.8	(1.20)	12.7	(1.09)	12.8	(0.87)	809		12.4	(1.26)	12.9	(1.27)	12.4	(1.26)	12.9	(1.19)	13.7	(1.67)
Maternal lifestyle at pregnancy registration																						
Current Smoking (+)	53	(6.5%)	4	(4.3%)	33	(5.4%)	9	(11.8%)	7	(22.6%)	44	(5.5%)	10	(9.0%)	24	(4.8%)	2	(2.6%)	6	(6.7%)	2	(8.0%)
Alcohol consumption (+)	65	(8.0%)	7	(7.6%)	50	(8.2%)	5	(6.7%)	3	(9.7%)	87	(10.9%)	9	(8.1%)	56	(11.3%)	6	(8.1%)	11	(12.2%)	5	(20.8%)
Eating habits: Skipping breakfast (+)	169	(20.8%)	15	(16.3%)	117	(19.1%)	25	(32.9%)	12	(38.7%)	168	(20.7%)	20	(17.9%)	101	(20.0%)	17	(21.8%)	23	(25.3%)	7	(26.9%)
Eating habits: Having afternoon snack (one or more times/day)	612	(76.0%)	71	(78.0%)	470	(77.3%)	50	(66.7%)	21	(67.7%)	603	(75.3%)	86	(78.9%)	371	(74.2%)	63	(81.8%)	66	(74.2%)	17	(65.4%)
Eating habits: Having midnight snack every day (+)	28	(3.7%)	4	(4.5%)	21	(3.6%)	2	(3.0%)	1	(3.3%)	20	(2.6%)	4	(3.8%)	10	(2.1%)	1	(1.3%)	3	(3.4%)	2	(8.3%)
Sleep status (Average, in a weekday)																						
Sleep duration (hours): means (SD)	805		7.4	(0.78)	7.4	(0.90)	7.3	(0.80)	6.9	(1.03)	805		7.36	(0.86)	7.34	(0.92)	7.5	(0.94)	7.25	(0.82)	7.0	(0.93)
Working (+)	362	(44.2%)	36	(38.7%)	272	(43.8%)	34	(45.9%)	20	(64.5%)	375	(46.3%)	42	(37.5%)	244	(48.4%)	39	(50.0%)	40	(44.4%)	10	(38.5%)
Paternal age (years): means (SD)	811		32.3	(5.72)	31.7	(5.29)	32.4	(4.72)	32.2	(5.37)	805		31.9	(5.96)	31.4	(5.22)	33.1	(4.84)	32.8	(5.21)	33.8	(5.53)
Paternal lifestyle at pregnancy registration																						
Current Smoking (+)	556	(68.1%)	57	(62.0%)	422	(68.3%)	48	(63.2%)	29	(93.5%)	545	(67.5%)	75	(67.0%)	339	(67.4%)	53	(67.9%)	58	(65.2%)	20	(76.9%)
Other family member’s lifestyle at pregnancy registration																						
Current Smoking (+)	615	(78.4%)	70	(76.9%)	468	(78.8%)	54	(77.1%)	23	(79.3%)	618	(79.1%)	84	(79.2%)	398	(81.6%)	55	(74.3%)	63	(71.6%)	18	(72.0%)

Abbreviations: SD, Standard deviation.

**Figure 1:**
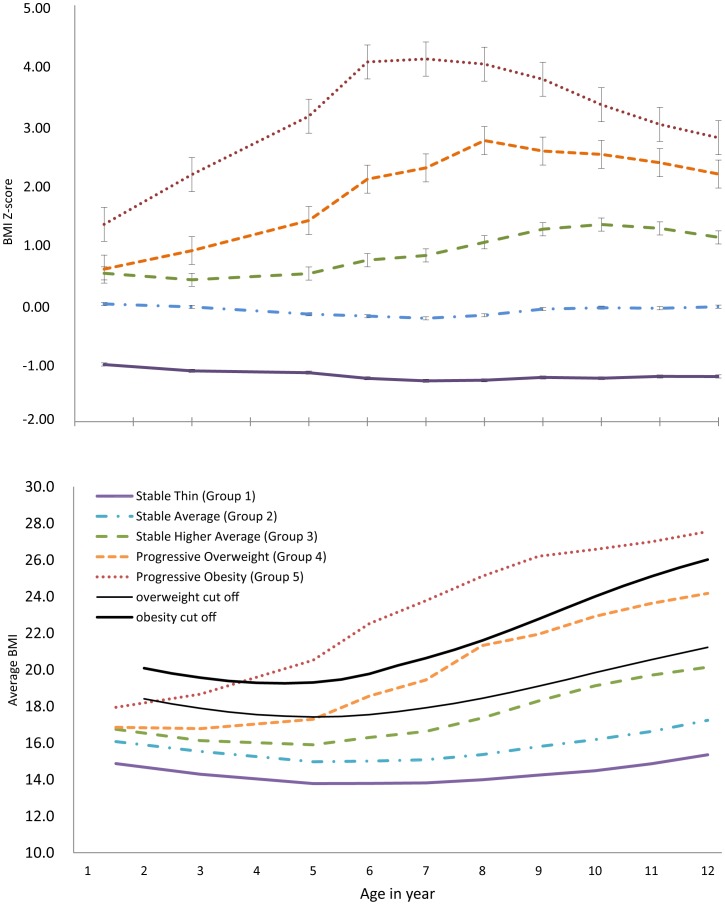
Trajectories of Body mass index (BMI) and the average BMI of boys aged 1.5 to 12 years in Koshu City, Japan, 1991–1998. Error bars indicate the standard error of the mean for each observed group. Group 1, “stable thin”; Group 2, “stable average”; Group 3, “stable high average”; Group 4, “progressive overweight”; Group 5, “progressive obesity.”

**Figure 2:**
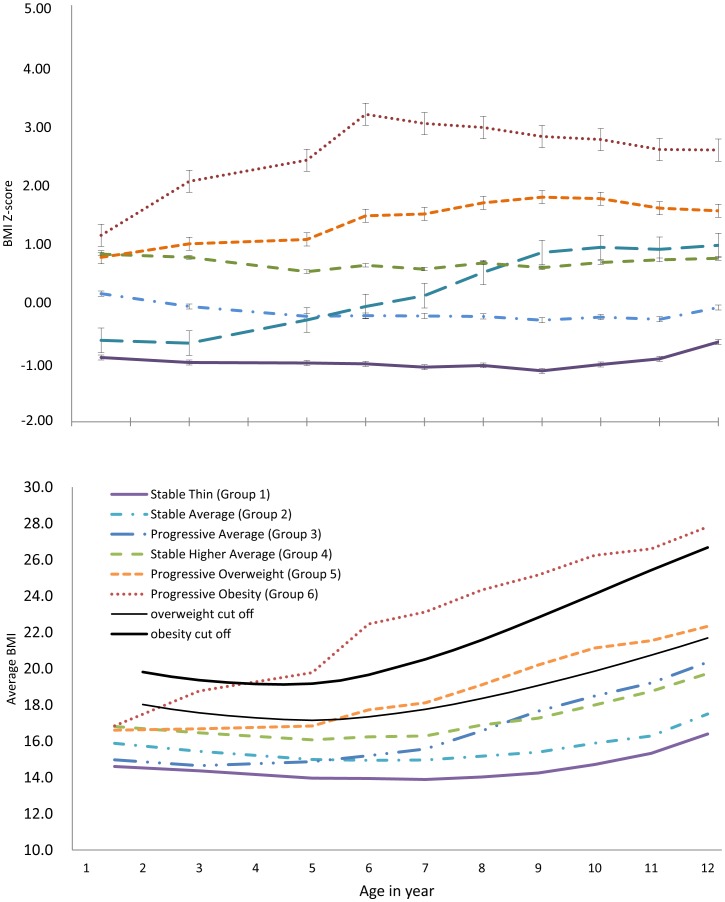
Trajectories of Body mass index (BMI) and the average BMI of girls aged 1.5 to 12 years in Koshu City, Japan, 1991–1998. Error bars indicate the standard error of the mean for each observed group. Group 1, “stable thin”; Group 2, “stable average”; Group 3, “progressive average”; Group 4, “stable high average”; Group 5, “progressive overweight”; Group 6, “progressive obesity.”

When modeling the BMI trajectory, the Bayesian Information Criterion (BIC) score increased as more groups were added. Therefore, based on clinical knowledge and the objectives of the analyses, a five-group model was selected for the boys and a six-group model for the girls (Figures 1 and 2). Among boys, 12.6% were categorized into Group 1, with an average BMI z score of −1.22 and an average BMI of 14.4 (Figure 1). This group maintained the lowest average BMI score throughout the developmental trajectory (Figure 1), and was, therefore, labeled the “stable thin” group. In this group, the average BMI gradually decreased until 7 years of age and then started to increase (Figure 1). The majority of boys in the study population were included in Groups 2 (42.2%) and 3 (30.5%). The average BMI z score was almost 0 throughout the trajectory for Group 2, was slightly larger, between 0.39 and 1.31, for Group 3. Group 2 was named “stable average” and Group 3 was referred to as “stable high average.” The average BMI of the boys in Group 4 (10.5%) exceeded the overweight threshold at age 5 and continued to rise throughout the observation period. This group was named the “progressive overweight” group. Group 5 (4.2%) had the highest BMI scores, exceeding the overweight threshold at around 2 years of age and surpassed the obesity threshold around 4 years of age; these individuals were in the “progressive obesity” group.

An identical 5 groups were described for girls. Groups 1, 2, 4, 5, and 6 were named as “stable thin,” “stable average,” “stable high average,” “progressive overweight,” and “progressive obesity,” respectively (Figures 2). Group 3, composed 12.1% of the girls, showed a unique pattern of gradually increasing BMI z scores from −0.68 at age 5 to 0.93 at age 10. Therefore, this was denoted as the “progressive average” group.

A sensitivity analysis using the alternative dataset that included the BMI scores calculated at birth did not alter the numbers or shapes of the observed trajectory patterns.

### Predictors of Membership within Each Trajectory

Among the factors evaluated at the time of pregnancy, the mother’s BMI, smoking habits, skipping of breakfast, and sleep duration, as well as paternal smoking were associated with differences in the BMI trajectory patterns among boys. The child’s year of birth, mother’s age, alcohol consumption, snacking habits, psychosocial and socioeconomic status (e.g., educational attainment), and paternal age were not associated with the observed trajectory patterns. Amongst the girls, only the mother’s age and BMI, as well as the father’s age were associated with the BMI trajectory patterns (Table 1). Univariate multinomial logistic regression revealed that, compared to the stable average or stable high average groups (Groups 2 or 3 for boys and Groups 2 or 4 for girls), a 1 unit increase in maternal BMI was associated with 1.22 (95% confidence interval [CI]: 1.09, 1.36) and 1.27 (95% CI: 1.13, 1.42) times higher likelihood of the child being included in the “progressive obesity” groups among boys and girls, respectively. Multivariate models adjusted for children’s birth year and BMI, and maternal age, BMI at the time of pregnancy registry, parity, and educational attainment showed that mothers who smoked (OR: 5.42; 95%CI: 1.89, 15.50) or skipped breakfast during pregnancy (OR: 3.50; 95% CI: 1.52, 8.08) were more likely to have boys in the “progressive obesity” group than in the stable average trajectory groups, independent of maternal BMI, maternal age, or educational attainment. Although the association between paternal smoking and boys’ trajectory patterns was statistically significant, the 95% CI was very wide (OR: 14.23; 95% CI: 1.89, 107.09) (Table 2). These associations were not shown among girls. We also created another model adjusting for BMI of children aged 1.5 years. The findings were chiefly the same as those obtained for models for adjusting for BMI at birth. However, some estimates could not be obtained as some values of the BMI at 1.5 years were missing.

**Table 2:** Odds ratios and confidence intervals for being categorized in the trajectory groups compared to average trajectory groups (stable average and stable high average) by baseline parental characteristics among children in Koshu City, Japan, 1991–1998: Result of Multinomial Logistic Regression.

Variables	Girls				Boys			
	Crude		Adjusted^a^		Crude		Adjusted^a^	
	OR	95% CI	OR	95% CI	OR	95% CI	OR	95% CI
Maternal age								
Stable thin	1.03	0.98–1.08			0.99	0.94–1.04		
Stable average	1.00				1.00			
Progressive average (girls only)	1.05	0.99–1.11						
Progressive overweight	1.07	1.01–1.12			1.05	0.99–1.11		
Progressive obesity	1.06	0.97–1.16			1.01	0.93–1.10		
Maternal body mass index								
Stable thin	0.82	0.74–0.91			0.87	0.78–0.96		
Stable average	1.00				1.00			
Progressive average (girls only)	1.09	1.00–1.20						
Progressive overweight	1.12	1.04–1.21			1.17	1.08–1.27		
Progressive obesity	1.27	1.13–1.42			1.22	1.09–1.36		
Maternal educational attainment (more than high school)								
Stable thin	0.68	0.34–1.38			1.14	0.67–1.94		
Stable average	1.00				1.00			
Progressive average (girls only)	0.95	0.53–1.71						
Progressive overweight	0.72	0.34–1.51			0.68	0.38–1.21		
Progressive obesity	0.53	0.19–1.51			0.74	0.31–1.75		
Maternal parity (first childbirth)								
Stable thin	0.81	0.54–1.23			1.06	0.68–1.65		
Stable average	1.00				1.00			
Progressive average (girls only)	0.86	0.54–1.38						
Progressive overweight	0.63	0.40–1.00			0.81	0.49–1.36		
Progressive obesity	0.61	0.27–1.40			1.47	0.73–2.97		
Child’s BMI at birth								
Stable thin	0.72	0.06–0.85			0.86	0.72–1.04		
Stable average	1.00				1.00			
Progressive average (girls only)	0.75	0.62–0.91						
Progressive overweight	1.01	0.85–1.21			0.93	0.75–1.14		
Progressive obesity	1.46	1.10–1.95			0.99	0.14–1.34		
Maternal lifestyle at pregnancy registration								
Current Smoking (+)								
Stable thin	1.98	0.92–4.26	1.87	0.71–4.95	0.80	0.28–2.32	0.57	0.16–1.97
Stable average	1.00				1.00			
Progressive average (girls only)	0.54	0.12–2.33	0.67	0.14–3.28				
Progressive overweight	1.43	0.57–3.59	1.89	0.63–5.67	2.37	1.09–5.16	1.80	0.72–4.53
Progressive obesity	1.74	0.39–7.79	1.75	0.19–15.99	5.14	2.07–12.81	5.42	1.89–15.5
Alcohol consumption (+)								
Stable thin	0.69	0.33–1.45	0.58	0.25–1.36	0.92	0.41–2.10	0.89	0.38–2.09
Stable average	1.00				1.00			
Progressive average (girls only)	0.69	0.29–1.67	0.82	0.33–2.06				
Progressive overweight	1.09	0.55–2.18	1.17	0.55–2.48	0.80	0.31–2.08	0.74	0.25–2.18
Progressive obesity	2.07	0.74–5.75	0.96	0.20–4.66	1.20	0.35–4.09	1.59	0.44–5.80
Eating habits: Skipping breakfast (+)								
Stable thin	0.87	0.51–1.48	0.99	0.53–1.82	0.83	0.46–1.49	0.70	0.37–1.34
Stable average	1.00		1.00		1.00		1.00	
Progressive average (girls only)	1.11	0.62–1.99	1.10	0.53–2.25				
Progressive overweight	1.35	0.80–2.28	1.44	0.79–2.65	2.08	1.24–3.50	2.02	1.08–3.78
Progressive obesity	1.47	0.60–3.60	2.09	0.66–6.67	2.68	1.27–5.68	3.50	1.52–8.08
Eating habits: Having afternoon snack (one or more times/day)								
Stable thin	1.30	0.79–2.15	1.23	0.70–2.19	1.04	0.61–1.77	1.25	0.70–2.24
Stable average	1.00		1.00		1.00		1.00	
Progressive average (girls only)	1.56	0.85–2.89	1.48	0.74–2.93				
Progressive overweight	1.00	0.60–1.67	0.97	0.55–1.71	0.59	0.35–0.98	0.54	0.30–0.97
Progressive obesity	0.66	0.29–1.51	0.65	0.23–1.85	0.62	0.28–1.34	0.52	0.23–1.22
Eating habits: Having midnight snack every day (+)								
Stable thin	1.85	0.57–6.00	1.75	0.40–7.71	1.25	0.42–3.74	0.71	0.15–3.29
Stable average	1.00		1.00		1.00		1.00	
Progressive average (girls only)	0.62	0.08–4.92	N/A^b^					
Progressive overweight	1.64	0.44–6.10	2.42	0.56–10.39	0.82	0.19–3.58	1.12	0.23–5.44
Progressive obesity	4.24	0.87–20.51	8.28	0.99–69.48	0.92	0.12–7.08	0.97	0.12–8.02
Sleeping duration (per 1 hour longer)								
Stable thin	1.03	0.82–1.29	1.11	0.84–1.45	1.08	0.84–1.38	1.16	0.88–1.54
Stable average	1.00		1.00		1.00		1.00	
Progressive average (girls only)	1.17	0.90–1.53	1.30	0.95–1.80				
Progressive overweight	0.89	0.69–1.15	0.97	0.73–1.30	0.87	0.66–1.15	0.87	0.63–1.20
Progressive obesity	0.69	0.44–1.08	0.85	0.29–2.48	0.55	0.37–0.83	0.56	0.35–0.89
Working (+)								
Stable thin	0.64	0.42–0.97	0.58	0.35–0.94	0.81	0.52–1.27	0.64	0.38–1.07
Stable average	1.00		1.00		1.00		1.00	
Progressive average (girls only)	1.07	0.66–1.72	1.09	0.63–1.90				
Progressive overweight	0.85	0.54–1.34	0.83	0.50–1.38	1.09	0.67–1.77	1.35	0.78–2.36
Progressive obesity	0.67	0.30–1.50	0.54	0.19–1.50	2.33	1.10–4.95	2.81	1.21–6.52
Paternal smoking (+)								
Stable thin	0.98	0.63–1.52	1.00	0.61–1.65	0.76	0.48–1.19	0.68	0.41–1.12
Stable average	1.00		1.00		1.00		1.00	
Progressive average (girls only)	1.03	0.62–1.71	1.05	0.59–1.86				
Progressive overweight	0.91	0.56–1.45	1.03	0.61–1.75	0.80	0.48–1.31	0.70	0.40–1.22
Progressive obesity	1.61	0.64–4.09	1.91	0.63–5.83	6.73	1.59–28.51	14.23	1.89–107.1
Other family member’s smoking (+)								
Stable thin	0.86	0.51–1.46	1.03	0.57–1.88	0.90	0.53–1.52	0.84	0.47–1.48
Stable average	1.00		1.00		1.00		1.00	
Progressive average (girls only)	0.65	0.37–1.16	0.63	0.33–1.19				
Progressive overweight	0.57	0.34–0.96	0.65	0.37–1.16	0.91	0.50–1.64	0.75	0.39–1.41
Progressive obesity	0.58	0.24–1.43	0.82	0.25–2.67	1.03	0.41–2.59	0.99	0.38–2.58

Abbreviations: BMI, body mass index; CI, confidence interval; OR, odds ratio.

a Adjusted for children’s birth year and BMI, and maternal age, BMI at the time of pregnancy registry, parity, and educational attainment.

b Because of small number, estimates for “eating midnight snack” are not presented.

## Discussion

The results of this study suggest that there are at least 5 distinct BMI trajectory patterns in Japanese boys and 6 among girls. Further, the BMI at the early stages of life (age = 1.5 years) was indicative, to some extent, of the subjects’ BMI at 12 years of age. This finding is similar to those of recent studies that show that adiposity in childhood is positively associated with that in adulthood [20], [21]. However, some trajectories did not show the same results. Among the boys with a BMI of 17, some maintained their “stable high average” BMI, whereas others developed a “progressive overweight” pattern. Among girls, the 3 heaviest trajectory patterns started from the same average BMI, which was approximately 16–17. Moreover, there was a unique “progressive average” pattern in a girl, whose BMI at 1.5 years of age was lower than the “stable average” pattern. Those patterns include the rapid and progressive development of obesity as well as the gradual movement into the overweight category. As hypothesized, a unique feature of this Japanese study was the identification of a stable thin pattern, which has never been identified in Western populations [11]–[13]. This study also showed that maternal BMI and some unfavorable behaviors during early pregnancy impact a child’s pattern of body mass development. Furthermore, the impact of these maternal characteristics appears to be different between boys and girls.

A study in the United States by Mustillo et al. followed 991 white children aged 9–16 years and identified 4 groups with different developmental trajectories, including a group developing obesity and then returning to normal BMI after the age of 12 and a group developing obesity after the age of 12 [12]. Another study conducted in the United States [11], examined the BMI trajectory of 1,739 white, black, and Hispanic children aged 2–12 years and identified 3 developmental trajectories. They also found a group that developed obesity in later years (after the age of 8). However, the study that was most comparable to the present study explored BMI trajectories of boys and girls, separately, in Canada. In this Canadian study, Hejazi et al. analyzed self-reported BMIs of 973 children aged 2–8 years and identified 3 BMI trajectories for boys and 4 for girls, including a pattern of declining BMI in later years and a J-shaped rising BMI pattern [13]. The present study, having advantages in terms of sample size, objective measurements of BMI, and study duration, found 2 additional patterns among Japanese children, although both studies were consistent in terms of identifying an additional pattern for girls. The existence of multiple normal to thin-weight patterns in Japan might reflect a lower BMI among Japanese children compared to Western children, potentially due to differences in dietary and cultural habits between the countries [15].

Our study and the Canadian study [11] both found that girls had more variation in their BMI trajectories than do boys, having the additional “progressive average” pattern among Japanese girls. This might be explained by the earlier development of secondary sex characteristics among girls, as the pubertal growth spurt usually occurs in conjunction with an increase in BMI. In Japan, 96% of girls develop secondary sex characteristics at the age of 12 or earlier. An alternative explanation for the observed gender differences may be the differential behavioral or lifestyle patterns between the sexes. Gender differences in social behavior and diet could also help to explain the observed gender differences in BMI trajectories [22]–[24]. For example, analyses of the present results revealed that mothers who regularly skipped breakfast during pregnancy contributed to the elevated risk of obesity in boys, but not girls. This suggests that the impact of maternal lifestyle on developmental patterns could differ by gender, potentially due to the impact of parent-child associations [18], [25], [26].

Typically, an adiposity rebound (the first increase in BMI after a nadir) happens around 5–6 years of age [27]. However, the present study suggested that the period of adiposity rebound might differ, based on the BMI trajectory pattern. That is, stably thin children may have an adiposity rebound that occurs both more slowly and later, around the age of 7 years. Those children categorized in the groups of progressive overweight and progressive obesity did not show a clear rebound in their adiposity, or the rebound may have occurred between 1.5–3 years; the period during which BMI information was not collected. Previous reports have suggested that the early occurrence of adiposity rebound may contribute to the risk of developing obesity in later years [28].

Potential determinants of physical developmental patterns can be categorized into genetic predisposition, the prenatal environment, and the postnatal environment [29]. The link found between maternal BMI and an overweight-type development pattern in the child supports the existence of the genetic or intrauterine effects. A growing body of epidemiologic and animal experimental evidence supports a link between *in utero* exposure to toxic substances or environmental conditions and the development of obesity in children, although the underlying mechanisms have not been completely elucidated [18], [30]–[34].

One potential limitation of the present study is that the number of groups and the shape of each group’s trajectory are not fully validated. However, our preliminary analysis using categories based on BMI trajectory (e.g., the “stable, thin” pattern includes those who have BMI z score of −1 or less at baseline and at the last survey) showed similar trends in the association between these patterns and their potential determinants including maternal BMI and smoking during pregnancy. This supports the validity of our analytical approach. Another potential limitation is the lack of certainty regarding its generalization to other regions of Asia, as the samples were only collected from a single region within Japan. Another potential limitation is the lack of detailed data on the physical development in utero (gestational weight gain) that could also affect the growth trajectories after birth. Moreover, the estimates based on our multivariate models may not be sufficiently adjusted for their potential measured and unmeasured confounders. We selected the covariate to be adjusted based on the theoretical consideration of confounding and the validity of statistical modeling (e.g., avoiding multicollinearity between variables). Although a 12-year longitudinal study period was an advantage of this study, further studies may require an even longer observation period with repeated measurements. Such a study would be particularly important in order to understand the independent and interactive impact of heredity and pre- and postnatal environments on BMI trajectories [35].

In conclusion, we found multiple trajectories of body mass development, which start to diverge early in life. Some modifiable factors were also identified, which could determine unfavorable trajectories. Based on data from this and other studies, BMI trajectories appear to vary across demographics, with gender and region being the main contributing elements. Data from this study support the concept that preventive interventions focused on the early development period, which target modifiable individual and environmental determinants, would likely be effective. A better understanding of the underlying mechanisms and determinants of BMI trajectory patterns are expected to make those interventions more effective.

## Materials and Methods

### Study Cohort

The analyses were based on data obtained through Project Koshu, a register-based prospective cohort study in Japan. The study population comprised all 1,644 children (825 boys and 819 girls) born between April 1991 and March 1998 in Koshu City, Japan, and their mothers. The expectant mothers were recruited at the beginning of their pregnancy, throughout Koshu City, where the local law requires registration of all new pregnancies. During pregnancy registration, a questionnaire on the lifestyles and the habits of the mothers and their children and families was administered to the mothers. During infant medical examinations, data were obtained regarding the infant’s growth and physical characteristics. As the children entered school, anthropometric data continued to be collected during annual measurements in each grade, as required by the School Health Law. Data of 1518 children (768 boys and 750 girls; 92.3%) who had been followed for 12 years, with at least 1 usable data point in their follow-up period, were analyzed. Three pairs of twins as well as participants who lacked baseline information on weight and height were excluded from the data analyses. Overall participation rates fell during the course of the study from 84.6% at 18 months of age to 74.9% by age 12.

### Measures

#### BMI of children

Data on the birth height and weight of the children in the study were obtained from the Maternal and Child Health Handbook. This record serves as an aid in monitoring child health and growth and is required to be provided to expectant mothers at the time of pregnancy registration [36]. Data on the height and body weight of the children were obtained from measurements taken during health checkups at ages 1.5, 3, and 5 and during annual school health monitoring for children aged 6–12 years. BMI scores were calculated using the standard formula: body weight (kg)/height (m)^2^. To maximize comparability, individual BMI z-scores were also calculated, as described in the World Health Organization standard [37]. Due to the unreliable nature of height measurements at birth, the BMIs at birth were not used in the primary analyses; they were, however, included in the sensitivity analyses to confirm the robustness of the data.

#### Maternal and familial variables

Although direct evidence regarding the determinants of trajectory patterns of childhood BMIs is lacking, some empirical studies have suggested that maternal health behaviors during pregnancy (smoking, alcohol consumption, eating habits, and sleep status), socioeconomic status, and maternal BMI scores impact a child’s weight [11], [12]. Therefore, in this study, the following factors were considered as independent variables having potential impact on the BMI trajectory patterns of children: maternal and familial smoking habits (smoking, had quit smoking, or never smoked), parental age, maternal BMI, maternal alcohol consumption (consuming alcohol, had stopped consuming alcohol, or never consumed alcohol), breakfast habits (having or skipping daily breakfast), snacking habits (having more than 1 per day or having 1 or fewer per day), average sleep duration, educational attainment (high school graduate or not having completed high school), and employment status (employed or unemployed). At the first pregnancy checkup, maternal height and weight during the first trimester were assessed by an obstetrician or midwife. The data were recorded in the Maternal and Child Health Handbook.

### Statistical Analyses

#### BMI trajectory patterns

BMI trajectories were determined by fitting a semiparametric mixture model, using the PROC TRAJ macro in SAS version 9.2 (SAS Institute, Cary, NC)[38]. We fitted this model to the data for eight BMI measures in children grouped by sex. This group-based modeling approach made it possible to identify a number of discrete classes, each having a specific intercept and age-slope with an estimated population prevalence [39]. Based on recent studies [40], cubic (third-order polynomial) shapes of the trajectories, the most flexible option available in the PROC TRAJ macro, were assumed. Estimation of trajectories was accomplished using the censored normal model, typically used to model the conditional distribution of censored variables where there is a cluster of data at the maximum or minimum values [40].

Following Nagin’s suggestions [39], the Bayesian Information Criterion [41] and the log of the Bayes factor [42] were used to find the optimal number of patterns in the BMI trajectories. Part of this analysis involved the identification of the point where the sign of the log of the Bayes factor changed. Nagin has recommended that if this BIC-based criterion does not clearly identify the number of patterns, i.e., the BIC continuously increases as more groups are added, more subjective criteria, based on domain knowledge and the objective of the analysis, should be considered [43].

#### Potential determinants of BMI trajectory patterns

To explore the factors determining the BMI trajectory patterns in the children, the basic statistics were described, and their crude associations with BMI trajectory patterns were tested using univariate multinomial logistic regressions. Then, multivariate multinomial logistic regressions were fitted to identify the independent impact of each factor on the children’s BMI trajectory patterns. These analyses were performed separately for boys and girls because of the gender differences in physical development [44]. All *P* values were two-tailed.

#### Ethics Statement

This study was approved by the Ethical Review Board of the University of Yamanashi, School of Medicine. A full description of the setting, sample, and data collection methods can be found elsewhere [18], [25], [45]. Informed assent for children was taken by self-reported questionnaires, and the parents and guardians were provided the opportunity to opt out of participation in this study.
